# Circadian Regulation of the Na^+^/K^+^-Atpase Alpha Subunit in the Visual System Is Mediated by the Pacemaker and by Retina Photoreceptors in *Drosophila Melanogaster*

**DOI:** 10.1371/journal.pone.0073690

**Published:** 2013-09-10

**Authors:** Milena Damulewicz, Ezio Rosato, Elzbieta Pyza

**Affiliations:** 1 Department of Cell Biology and Imaging, Institute of Zoology, Jagiellonian University, Kraków, Poland; 2 Department of Genetics, University of Leicester, Leicester, United Kingdom; Karlsruhe Institute of Technology, Germany

## Abstract

We investigated the diurnal oscillation in abundance of the catalytic α subunit of the sodium/potassium pump (ATPα) in the brain of *Drosophila melanogaster*. This rhythm is bimodal and is particularly robust in the glia cells of the lamina, the first optic neuropil. We observed loss of ATPα cycling in lamina glia in behaviourally arrhythmic *per^01^* and *tim^01^* mutants and in flies overexpressing the pro-apoptotic gene *hid* in the PDF-positive clock neurons. Moreover, the rhythm of ATPα abundance was altered in *cry^01^* and *Pdf^0^* mutants, in flies with a weakened clock mechanism in retina photoreceptor cells and in those subject to downregulation of the neuropeptide ITP by RNAi. This complex, rhythmic regulation of the α subunit suggests that the sodium/potassium pump may be a key target of the circadian pacemaker to impose daily control on brain activities, such as rhythmic changes in neuronal plasticity, which are best observed in the visual system.

## Introduction

In most organisms an endogenous circadian clock underpins daily rhythms in biochemistry, physiology and behavior. In animals, a relatively small number of neurons constitute the so-called central clock or circadian pacemaker that transmits rhythmic information to target organs and tissues by electric and/or chemical signals, such as neurotransmitters and hormones. In the fruit fly *Drosophila melanogaster* the central clock is composed of 150 neurons divided into Lateral Neurons (LNs) – subdivided into dorsal (LN_d_s), small ventral (s-LN_v_s) large ventral (l-LN_v_s) and posterior (LPNs) neurons - and three groups of Dorsal Neurons (DNs) called DN_1_s, DN_2_s and DN_3_s. The molecular mechanisms of the circadian clock are based on the cyclic expression of two core clock genes, *period* (*per)* and *timeless* (*tim)*. The key steps are centered around the PER and TIM proteins, which inhibit their own transcription only after activation through extensive phosphorylation that also targets them for degradation, resulting in a main rhythmic negative feedback loop to which others are interlocked [1]. Another fundamental property of the clock is entrainment to LD cycles, which is largely dependent upon CRY, a blue-light sensitive protein encoded by the *cryptochrome* (*cry*) gene. CRY activation and degradation are inter-dependent phenomena, triggered by exposure to light. Active CRY is responsible for initiating light-driven degradation of TIM, which also accelerates the turnover of PER [2], [3]. Finally, rhythmic information is exchanged among circadian neurons and then passed on to target tissues; although the molecular mechanisms are largely unknown neuropeptides seem to contribute to this process. The LNs and DNs synthesize several peptides that appear to transmit circadian information. Among them are the PIGMENT DISPERSING FACTOR (PDF), which is made by the LN_v_s [4], [5] with the exception of the so-called 5^th^ s-LN_v_, which, along with one LN_d_, produce the ION TRANSPORT PEPTIDE (ITP) instead [6], [7]. These peptides may modulate the physiology of target cells by changing the activity of enzymes, transporters, channels or pumps. For instance, after binding to PDF-R, a G protein coupled receptor, PDF leads to the activation of adenylate cyclase thus increasing cAMP levels in many pacemaker cells [8]–[12]. In contrast, the receptor for ITP and its molecular functions are unknown.

We have previously demonstrated that the first neuropil of the optic lobe, the lamina, is a site of pronounced circadian plasticity and we have described rhythmic morphological changes occurring in interneurons and glia [13]–[17]. These rhythms depend on the expression of clock genes in LNs, in some glial cells and in the retina photoreceptors [16], [18] and additionally on lamina neurotransmitters [19], [20]. We have also found that the α subunit of the *Drosophila* Na^+^/K^+^-ATPase (ATPα) may be involved in regulating circadian rhythms in the lamina. The abundance of this protein is under circadian modulation, which is dependent upon PER [21].

Here we used immunofluorescence and confocal microscopy to further investigate the rhythmic abundance of ATPα in the lamina and its function, and found that those rhythms depend on CRY and on peptidergic clock neurons producing PDF and ITP.

## Materials and Methods

### Animals

The following strains of *Drosophila melanogaster* were used: wild-type Canton S, *cry^01^* (null mutant of the clock gene *cryptochrome*) [22], *Pdf^0^* (null mutant of the clock neuropeptide *Pigment dispersing factor* gene) [5], *per^01^* (null mutant of the clock gene *period*) [23], *tim^01^* (null mutant of the clock gene *timeless*) [24], and several GAL4 lines: *cry*-*GAL4* [25], *Pdf*-*GAL4* [26], *repo*-*GAL4* [27], *gmr*-*GAL4* [28] – expressing the yeast transcription factor GAL4 under control of *cry*, *Pdf* , *repo* and *gmr* promoters, respectively. In addition we used: UAS-*dicer2*;;UAS-*itp-RNAi/*MKRS expressing *dicer2* (catalyzing the first step of RNA interference) and interfering RNA for *itp* under the control of UAS sequences [29], UAS-*cry-RNAi*/Cy0 expressing interfering RNA for *cry* [30], UAS-*hid* expressing the pro-apoptotic gene *hid* [31] and UAS-*Δcyc* expressing a dominant negative form of CYC [32]. For the rescue experiments we used: *cry*-*GAL4*/*UAS*-*cry*; *cry^01^*, and *Pdf*-*GAL4*/*UAS*-*Pdf* ; *Pdf^0^* [2], [5], [25]. Flies were maintained on standard cornmeal medium under 12 h of light and 12 h of darkness (LD12:12) conditions and at a constant temperature of 24°C. To downregulate ITP expression in clock neurons *cry*-*GAL4* males were crossed to UAS-*dicer2;;*UAS-*itp-RNAi* females. *Pdf*-GAL4 males were crossed to UAS-*hid* females to induce apoptosis in the PDF-positive cells.

### Immunohistochemistry

7 day old males were decapitated at ZT1, ZT4, ZT13 and ZT16 (ZT is *Zeitgeber* Time, where ZT0  =  lights-on and ZT12  =  lights-off) under LD conditions or at CT1, CT4, CT13 and CT16 (CT is Circadian Time, the subjective time under constant conditions with CT0 =  subjective lights-on and CT12 =  subjective lights-off) under constant darkness (DD). Approximately 30 flies were used for each time point and every experiment was repeated at least three times. Heads were fixed in 4% paraformaldehyde in phosphate buffer saline (PBS; pH 7.4) for 4 h (all procedures were carried out at room temperature unless otherwise stated), then washed twice in PBS, cryoprotected by incubation in 12.5% sucrose for 10 min and then in 25% sucrose at 4°C overnight. Heads were then embedded in Tissue-Tek, frozen in liquid nitrogen, and sectioned (20 µm thickness) on a cryostat. The sections were washed in PBS for 30 min and then 5 times in phosphate buffer with added 0.2% Triton X 100 (PBT). Afterwards, they were incubated in a mix of 5% Normal Goat Serum (NGS) and 0.5% Bovine Serum Albumin (BSA) for 30 min. Mouse α5 primary antibodies against chicken Na^+^/K^+^-ATPase α-subunit (but also specific for *Drosophila* [33]), obtained from the Developmental Studies Hybridoma Bank, were added to the mix (diluted 1∶50) and incubated for 24 h at 4°C. The sections were then washed 6 times in PBT/BSA, blocked in 5% NGS for 45 min and incubated with Cy3 conjugated goat anti-mouse secondary antibody (Jackson Immuno Research, diluted 1∶500), overnight at 4°C. After a series of washes (twice in BSA, six times in PBT, and twice in PBS) the sections were mounted in Vectashield medium (Vector) and examined with a Zeiss Meta 510 Laser Scanning Microscope. Confocal images of the lamina were captured at 1 μm intervals, 6 μm Z-stacks were analysed.

### Quantitative comparison of immunofluorescence values

To measure the immunofluorescence intensity of glial cells in the lamina, we analysed confocal images of frontal sections. The acquisition parameters were maintained constant for all preparations. We randomly selected three areas on each 16-bit confocal image and, using ImageJ software (NIH, Bethesda), we calculated the fluorescence intensity for each image. This is represented by the Mean Gray Value (the sum of the gray values of all pixels in the area divided by the number of pixels within the selection) of each area averaged across the three areas. For each fly the fluorescence intensity measures were then averaged across all images representing the same time point, obtaining mean levels of fluorescence intensity. These were then normalized across time points using ZT1 or CT1 as reference (set to 1). Thus, for each time point we obtained a fluorescence index that was invariably 1 for ZT1 and CT1. The fluorescent indices for different time points were compared by ANOVA followed by Tukey's post-hoc tests (p<0.05) using Statistica software.

## Results

**Figure 1:**
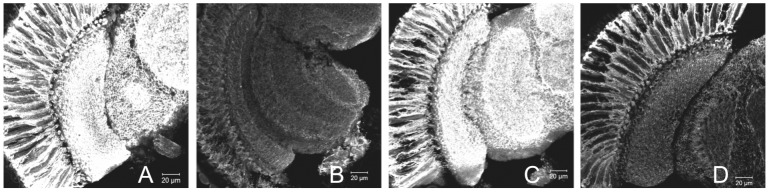
α5 immunolabeling of Na^+^/K^+^-ATPase α-subunit (ATPα) in the optic lobe of CantonS flies at specific time points under LD 12:12. The intensity of immunofluorescence in the lamina differs at different time points: A – ZT1, B – ZT4, C – ZT13, D – ZT16. The fluorescence signal is maximal in the lamina and in the medulla neuropils.

**Figure 2:**
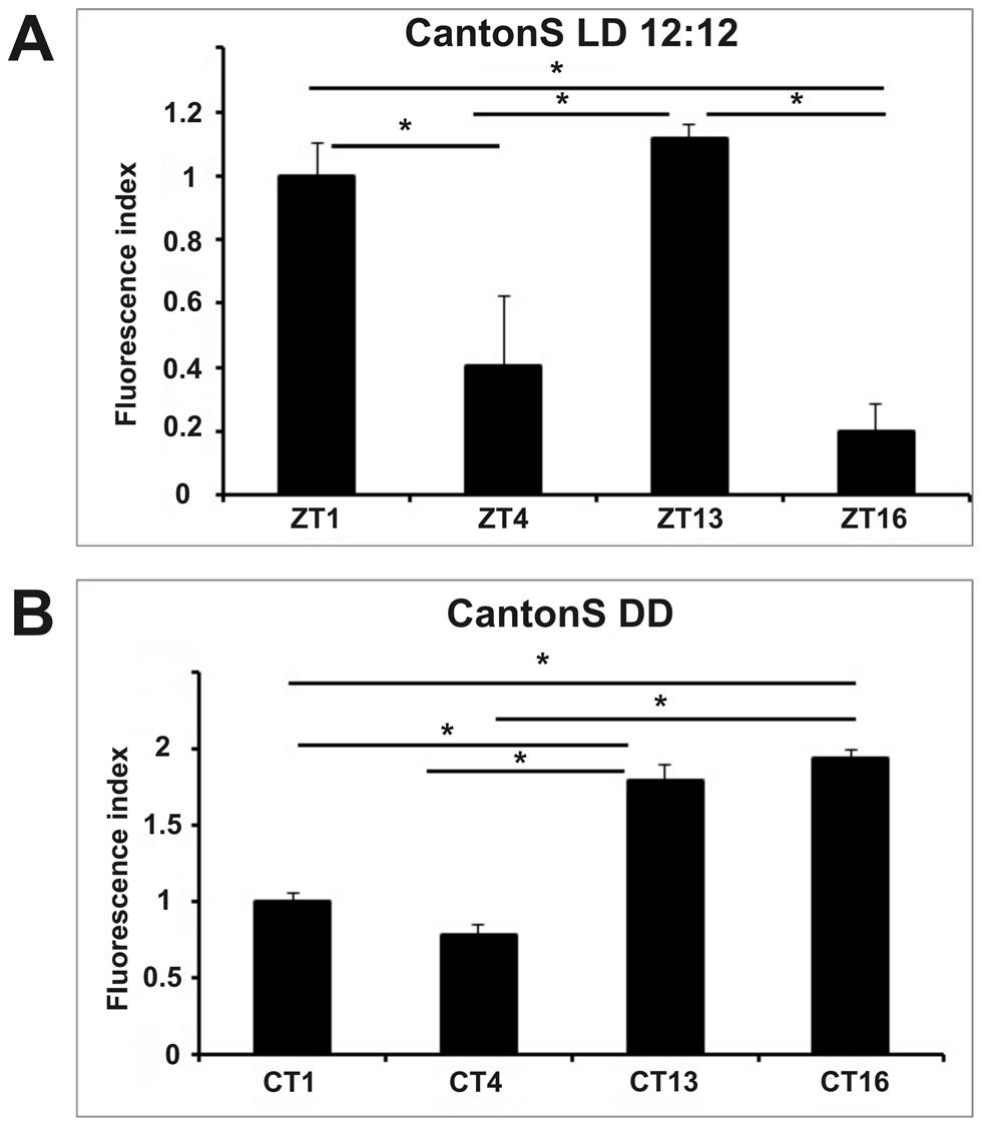
Rhythmic immunoreactivity of ATPα in wild-type CantonS flies under LD 12:12 (A) and DD (B). The fluorescence index ± SE is shown as a function of time. Under LD 12∶12 statistically significant differences were detected between ZT1 and ZT4, ZT1 and ZT16, ZT13 and ZT4, ZT13 and ZT16. The fluorescence index was highest at ZT13 and then lowered by 10.4% at ZT1, 63.7% at ZT4 and 82% at ZT16. In DD the fluorescence index was significantly higher during the subjective night (CT16 and CT13), than the subjective day (48.4% reduction at CT1 and 59.8% reduction at CT4). Parametric ANOVA Tukey's test; p<0.05.

**Figure 3:**
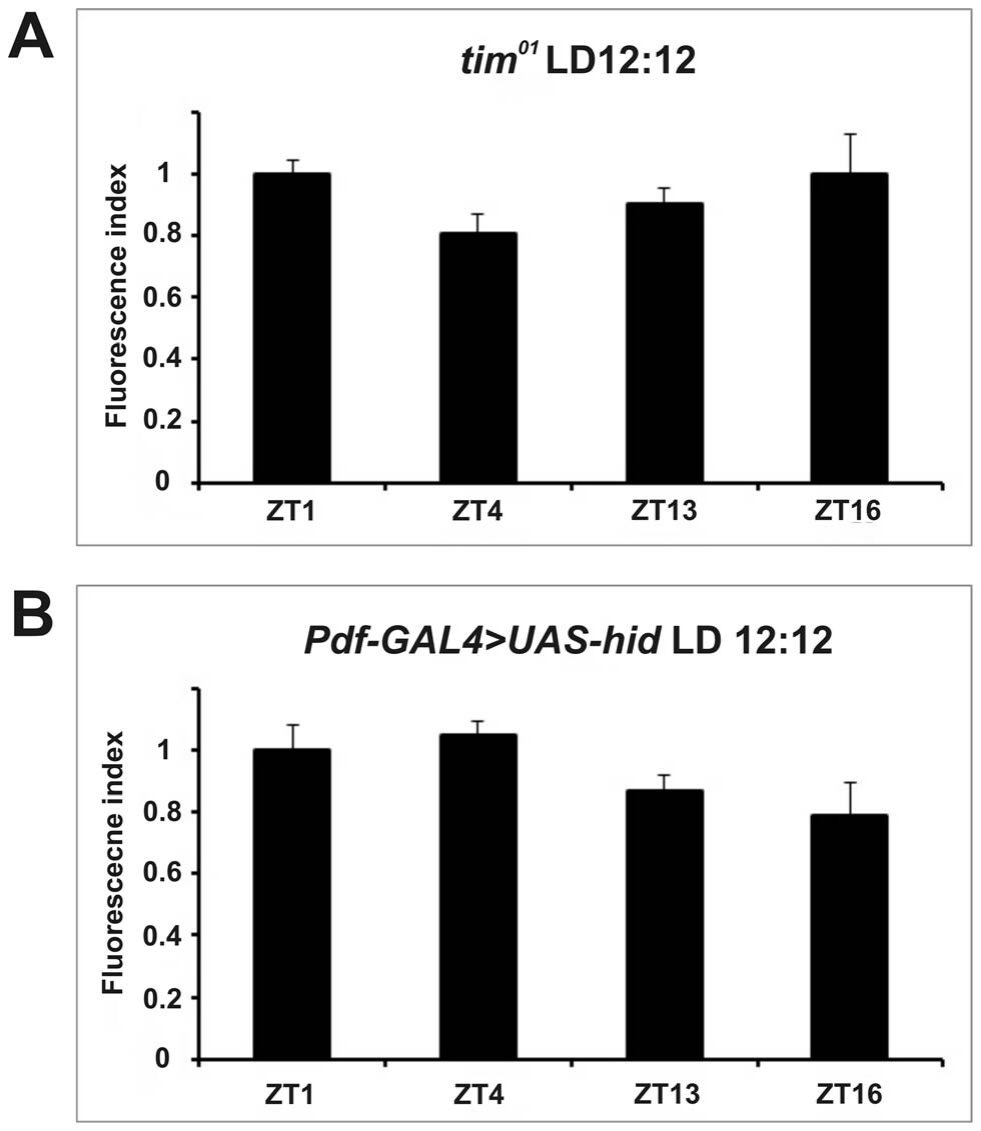
Arrhythmic ATPα immunoreactivity in mutants that affect the clock (A) or the viability of clock cells (B). The fluorescence index ± SE is shown as a function of time (A) *tim^01^*, (B) *Pdf-GAL4> UAS-hid* flies. There are not statistically significant differences between time points. Parametric ANOVA Tukey's test; p<0.05.

We immunolabeled the α subunit of the *Drosophila* Na^+^/K^+^-ATPase using anti-ATPα (anti-α5) primary and fluorescent secondary antibodies. Using confocal microscopy applied to cryosections of the lamina, we measured the intensity of the immunofluorescence signal at different time points (Figure 1). In wild-type (Canton S) flies fluorescence was significantly more intense at the beginning of both day (ZT1) and night (ZT13) compared to the other time points (ZT4 and ZT16, ANOVA with Tukey's post-hoc comparisons, Figures 1, 2A), in accordance with our previous results [21]. We observed that in the lamina expression of the α subunit is particularly pronounced in glia, confirming cyclic expression in those cells. The rhythm in immunoreactivity appeared under control of the circadian clock as it was not detected in null mutants of the clock genes *per^01^* [21] and *tim^01^* (Figures 3A). However, it was additionally modulated by the LD cycle as in wild type flies the rhythm adjusted to a unimodal profile under constant conditions (DD, Figure 2B). In particular, the intensity of the immunostaining was significantly higher during the subjective night (CT13 and CT16) than during the subjective day (CT1 and CT4). Moreover, the amplitude of the oscillation (measured as the ratio between the highest and the lowest records at the different time points) was about 30% higher in DD compared to LD (Table 1).

**Table 1:** Amplitude (measured as the ratio between the highest and the lowest records at the different time points) of ATPα immunoreactivity rhythms in all strains used in the study.

strain	amplitude
CantonS LD 12∶12	0.9±0.06
CantonS DD	1.2±0.01
*cry^01^* LD 12∶12	0.8±0.2
*cry^01^* DD	1.5±0.01
*cry* rescue	0.8±0.04
*Pdf^0^* LD 12∶12	0.6±0.04
*Pdf^0^* DD	1.1±0.2
*Pdf* rescue	0.8±0.03
*gmr-GAL4>UAS-cry-RNAi*	0.7±0.2
*repo-GAL4>UAS-cry-RNAi*	0.3±0.01
*gmr-GAL4>UASΔcyc*	0.6±0.05
*repo-GAL4>UAS-Δcyc*	0.5±0.1
*cry-GAL4>UAS-dicer2;UAS-itp-RNAi* LD 12:12	0.5±0.02
*cry-GAL4>UAS-dicer2;UAS-itp-RNAi* DD	1.6±0.08

**Figure 4:**
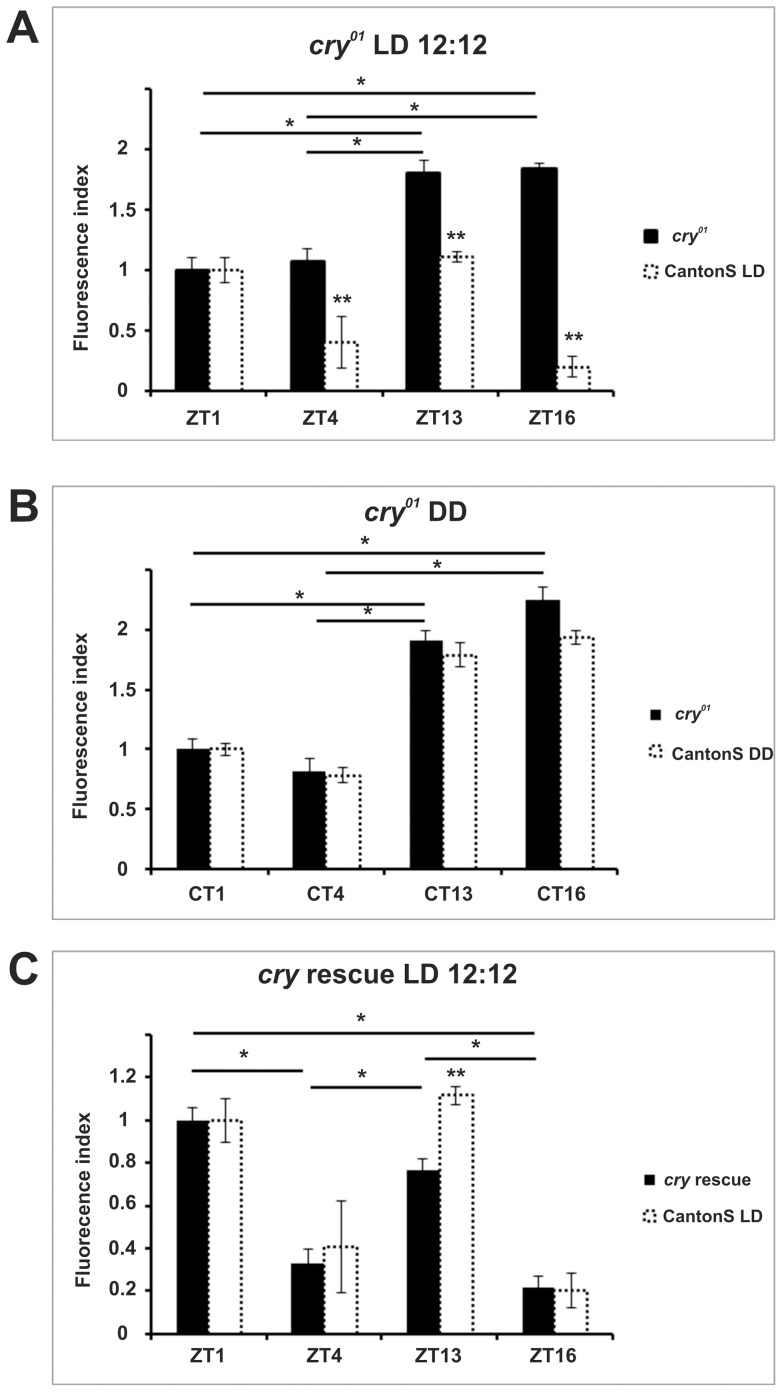
Pattern of ATPα immunoreactivity in *cry^01^* mutants under LD (A) and DD (B) and in rescue flies under LD (C). The fluorescence index ± SE is shown as a function of time. (A) *cry^01^* mutants under LD. ZT1 and ZT4 are significantly different from the other time points. The immunoreactivity of the α subunit was highest at ZT16 and decreased at other time points (ZT13 by 16%, ZT1 by 50.08%, and ZT4 by 56.4%). (B) *cry^01^* mutants under DD. The pattern of immunoreactivity was similar to LD conditions. The highest level of immunoreactivity was reached at CT16 and decreased by 15.3%, 55.8% and 63.7% at CT13, CT1 and CT4, respectively. (C) *cry* rescue under LD. Peak immunofluorescence was at ZT1 and then decreased by 70%, 20% and 82% at ZT4, ZT13 and ZT16, respectively. There are statistically significant differences between ZT1 and ZT4, ZT1 and ZT16, ZT13 and ZT4, ZT13 and ZT16. Parametric ANOVA Tukey's test; p<0.05. The two stars symbols indicate statistically significant differences between the experimental strains and CantonS controls at different time points.

Homozygous flies for the *cry^01^* allele [22] totally lack the blue-light photopigment CRYPTOCHROME (CRY), which has been implicated in circadian photoreception [2], [3], [34]. These mutants showed a unimodal rhythm of immunoreactivity under LD (with ZT13 and ZT16 significantly higher than ZT1 and ZT4) (Figure 4A) and DD (CT13 and CT16 higher than CT1 and CT4) (Figure 4B), which resembled the profile of wild-type flies under DD. We then used the GAL4/UAS system [35] to restore CRY expression in *cry^01^* flies (*cry-GAL4*>*UAS-cry, cry^01^*), reverting the profile of α-subunit immunoreactivity to bimodality (Figure 4C). To prove the specific nature of the rescue we showed that the *cry-GAL4* driver did not affect, *per se*, the rhythm (Figure S3B). These results suggest that CRY is required for the light-dependent modulation of α-subunit cycling in the lamina.

**Figure 5:**
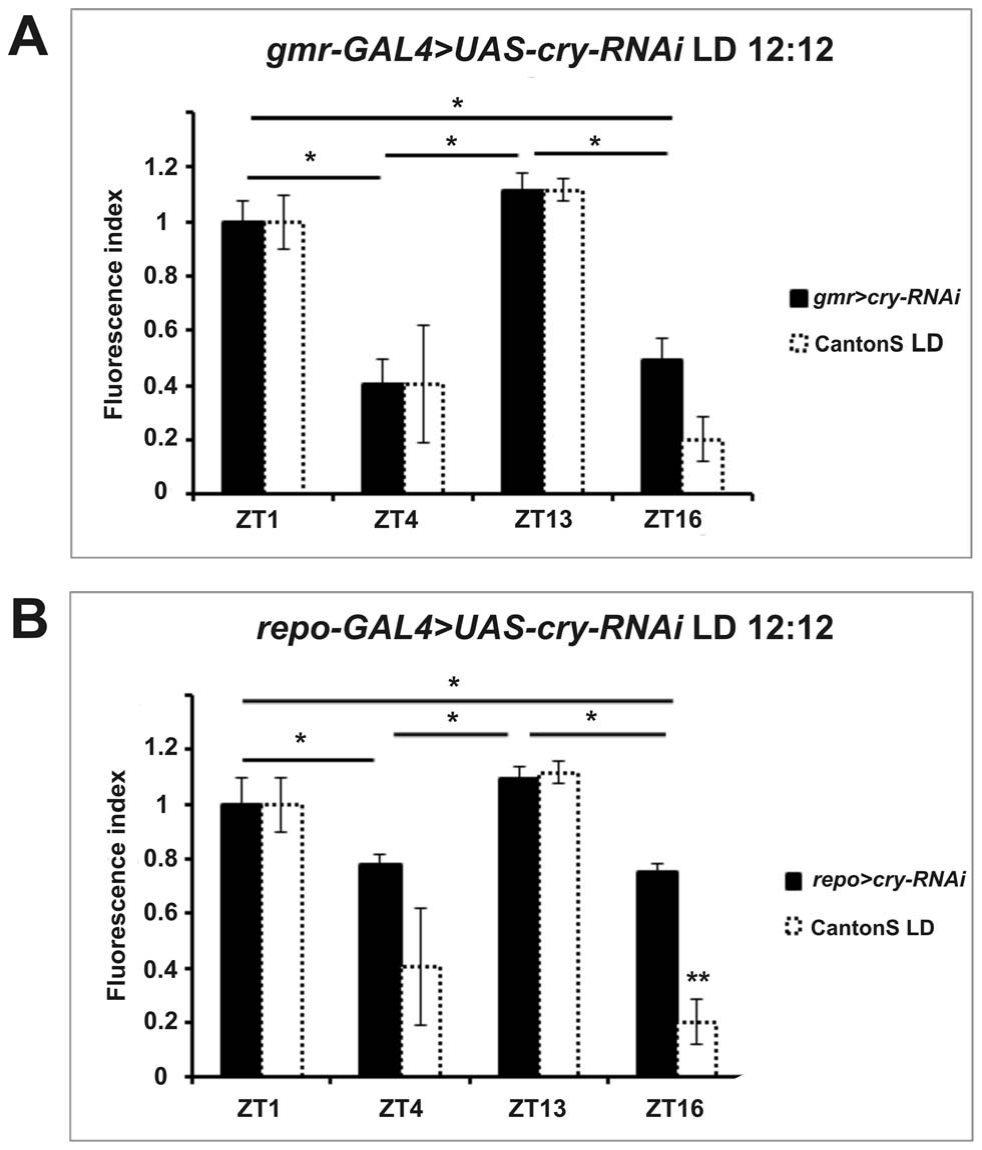
Pattern of ATPα immunoreactivity in flies with reduced CRY expression in photoreceptors (*gmr-GAL4>UAS-cry-RNAi*) (A) and glia (*repo-GAL4>UAS-cry-RNAi*) (B) under LD. The fluorescence index ± SE is shown as a function of time. (A) In *gmr-GAL4>UAS-cry-RNAi* flies the higher levels of immunoreactivity were observed at ZT1 and ZT13. The immunosignal decreased by 66.3% at ZT4 and by 58.3% at ZT16. Statistically significant differences were observed between ZT1 and ZT4, ZT1 and ZT16, ZT13 and ZT4, ZT13 and ZT16. (B) In *repo-GAL4>UAS-cry-RNAi* flies higher immunosignal was observed at ZT1 and ZT13 and then decreased by about 30% in ZT4 and ZT16. There were statistically significant differences between ZT1 and ZT4, ZT1 and ZT16, ZT13 and ZT4, ZT13 and ZT16. Parametric ANOVA Tukey's test; p<0.05. The two stars symbols indicate statistically significant differences between the experimental strains and CantonS controls at different time points.

**Figure 6:**
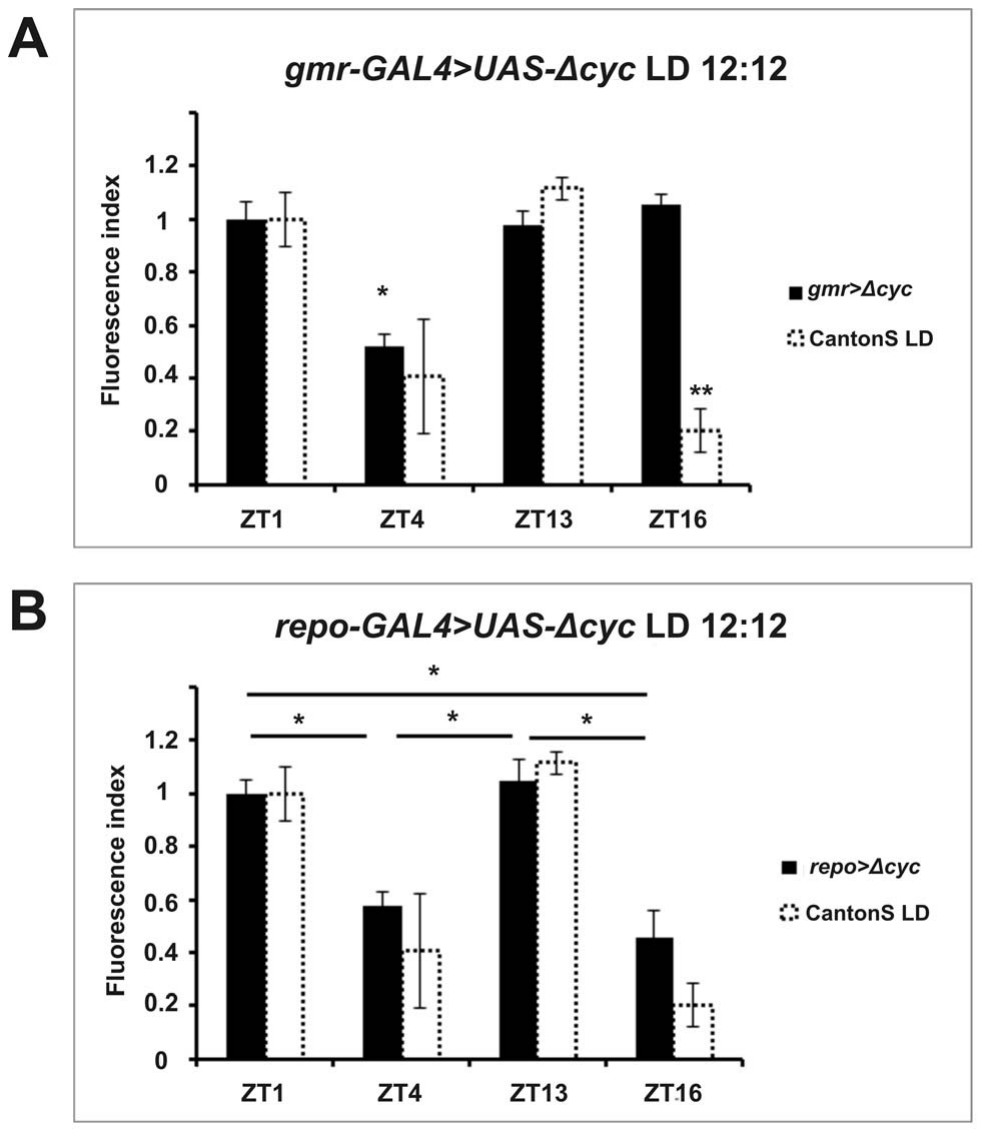
Pattern of ATPα immunoreactivity in flies with reduced clock activity in photoreceptors ( *gmr-GAL4>UAS-Δcyc*) (A) and glia (*repo-GAL4>UAS-Δcyc*) (B). The fluorescence index ± SE is shown as a function of time. (A) In *gmr-GAL4>UAS-Δcyc* flies immunofluorescence levels were high at ZT1, ZT13 and ZT16 and lowered by about 50% at ZT4. There were statistically significant differences between ZT4 and the other time points. (B) In *repo-GAL4>UAS-Δcyc* flies the immunofluorescence index was higher at ZT1 and ZT13 and lowered by 45.1% at ZT4 and by 56.4% at ZT16. There were statistically significant differences between ZT1 and ZT4, ZT1 and ZT16, ZT13 and ZT4, ZT13 and ZT16. Parametric ANOVA Tukey's test; p<0.05. The two stars symbols indicate statistically significant differences between the experimental strains and CantonS controls at different time points.

We then investigated whether cyclic, light-modulated α-subunit immunoreactivity rested upon cell-autonomous or non-autonomous functions of CRY. We adopted again the GAL4/UAS system and used RNA interference (RNAi) to downregulate CRY (*UAS-cry-RNAi*) in glia (*repo-GAL4*) or in all photoreceptor cells (*gmr-GAL4*) which, with the exception of R7 and R8, directly project to the lamina [36]. Using quantitative reverse-transcription PCR, we verified the effectiveness of RNAi in *gmr-GAL4>UAS-cry-RNAi* flies. These had 36% less *cry* transcript than *gmr-GAL4* controls (Figure S1). Under LD downregulation of *cry* in glia or photoreceptors resulted in bimodal cycling of α-subunit immunoreactivity, with ZT1 and ZT13 showing higher immunofluorescence than ZT4 and ZT16, analogous to the wild-type (Figure 5A, B). However, the amplitude of cycling was reduced when using the *repo-GAL4* driver perhaps suggesting that the cell autonomous functions of CRY are required to achieve wild- type regulation and produce the characteristic LD bimodality. We also used the same drivers to over-express CYCΔ, a 17 amino acid deletion of CYCLE (CYC) [32] (Figure 6A, B). The transcription factor CYC forms a heterodimer with CLOCK (CLK) to drive transcription of the main circadian genes involved in several negative feedback loops [37]. CYCΔ retains the protein dimerization domain but lacks the DNA binding region. Thus it acts as a dominant negative protein antagonizing CLK-CYC mediated transcription and blocking the cell-autonomous mechanisms of the clock. Expression of CYCΔ in glia (*repo-GAL4*>*UAS-cyc*Δ) increased the intensity of the immunofluorescence signal at ZT4 and ZT16, reducing the amplitude of its oscillation (Figure 6B, Table 1), but did not change the bimodal LD profile compared to wild type. Blocking the clock mechanism in photoreceptor cells (*gmr-GAL4*>*UAS-cyc*Δ) had, instead, a more profound effect resulting in lower amplitude cycling and alteration of the expression profile such that a significant reduction in immunofluorescence was only reached at ZT4 (Figure 6A). These results suggest that rhythmic inputs are more important than the endogenous feedback loop in determining cyclic expression of the Na^+^/K^+^-ATPase α-subunit in glia cells of the lamina.

**Figure 7:**
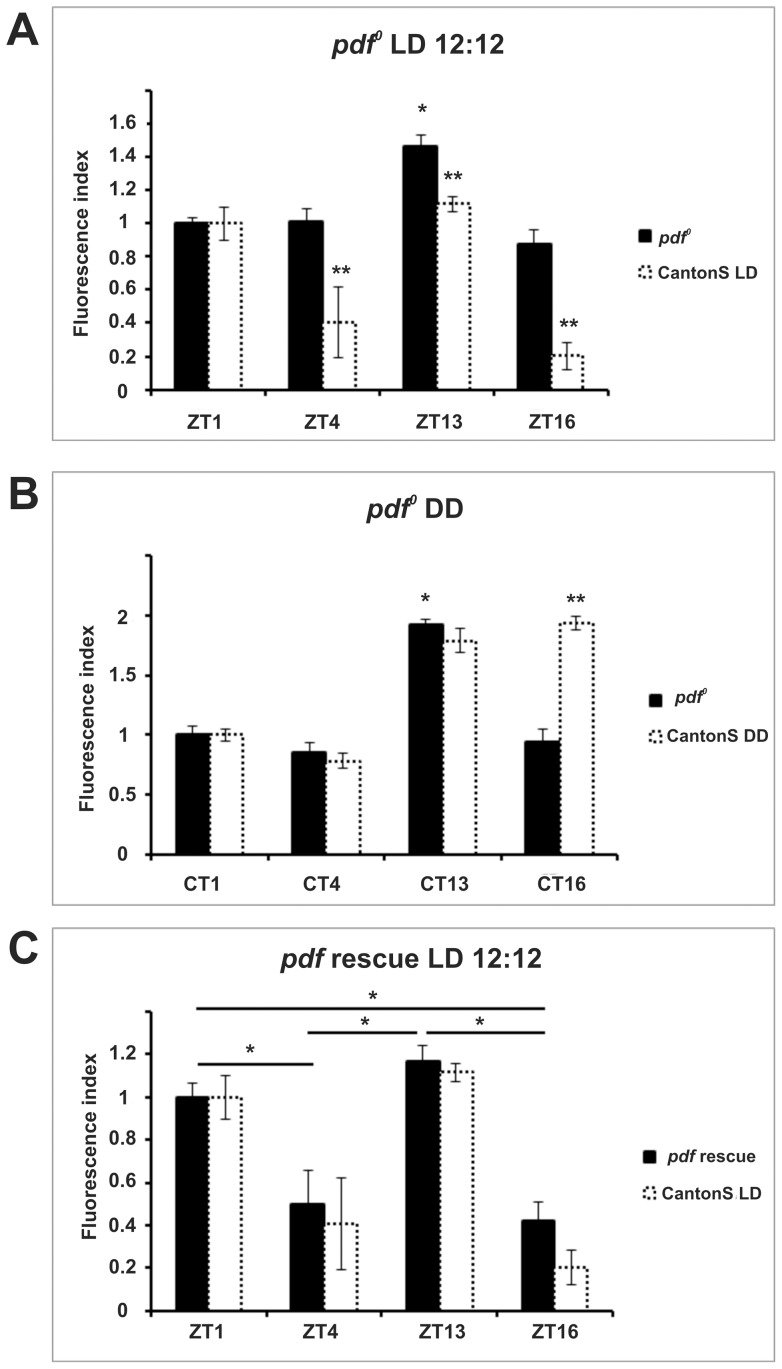
Pattern of ATPα immunoreactivity in *Pdf^0^* mutants under LD (A) and DD (B) and in rescue flies under LD (C). The fluorescence index ± SE is shown as a function of time. (A, B) In *Pdf^0^* mutants under both LD and DD conditions, the highest levels of immunofluorescence were observed at ZT13. At other time points the levels lowered by about 30% in LD and by about 50% in DD. There were statistically significant differences between ZT13 and the other time points under both LD and DD conditions. (C) In *Pdf* rescue flies the highest immunofluorescence level was at ZT13 that lowered by 15.6% at ZT1, by 57.6% at ZT4 and by 66.1% at ZT16. Statistically significant differences were seen between ZT1 and ZT4, ZT1 and ZT16, ZT13 and ZT4, ZT13 and ZT16. Parametric ANOVA Tukey's test; p<0.05. The two stars symbols indicate statistically significant differences between the experimental strains and CantonS controls at different time points.

In addition to photoreceptors, rhythmic signals may reach the lamina in the form of neuropeptides such as PDF and ITP, which are produced by clock neurons [11], [7]. Thus, we next tested the outcome of altering their regulation. Removing PDF signaling by means of a *Pdf*-null (*Pdf^0^*) mutant (Figure 7A, B) or by inducing cell death in PDF-expressing neurons (*Pdf-GAL4*>*UAS-hid*) (Figure 3B) seriously hampered rhythmicity under LD (Figures 7A and 3B). This was especially true for the latter genotype, which showed no sign of rhythmic immunolabeling, suggesting additional PDF-independent regulation from the LN_v_s. The *Pdf^0^* mutants showed a modest but still significant increase in fluorescence limited to ZT13 (Figure 7A). The same profile persisted under DD (Figure 7B). The restoration of PDF expression (*Pdf-GAL4>UAS-Pdf, Pdf^0^*) rescued the bimodal pattern characteristic of LD, and its amplitude (Figure 7C). These results show that PDF signaling, although not necessary for rhythmicity in DD, is required for maintaining high levels of α-subunit immunoreactivity at CT16 and for bimodal expression under LD.

**Figure 8:**
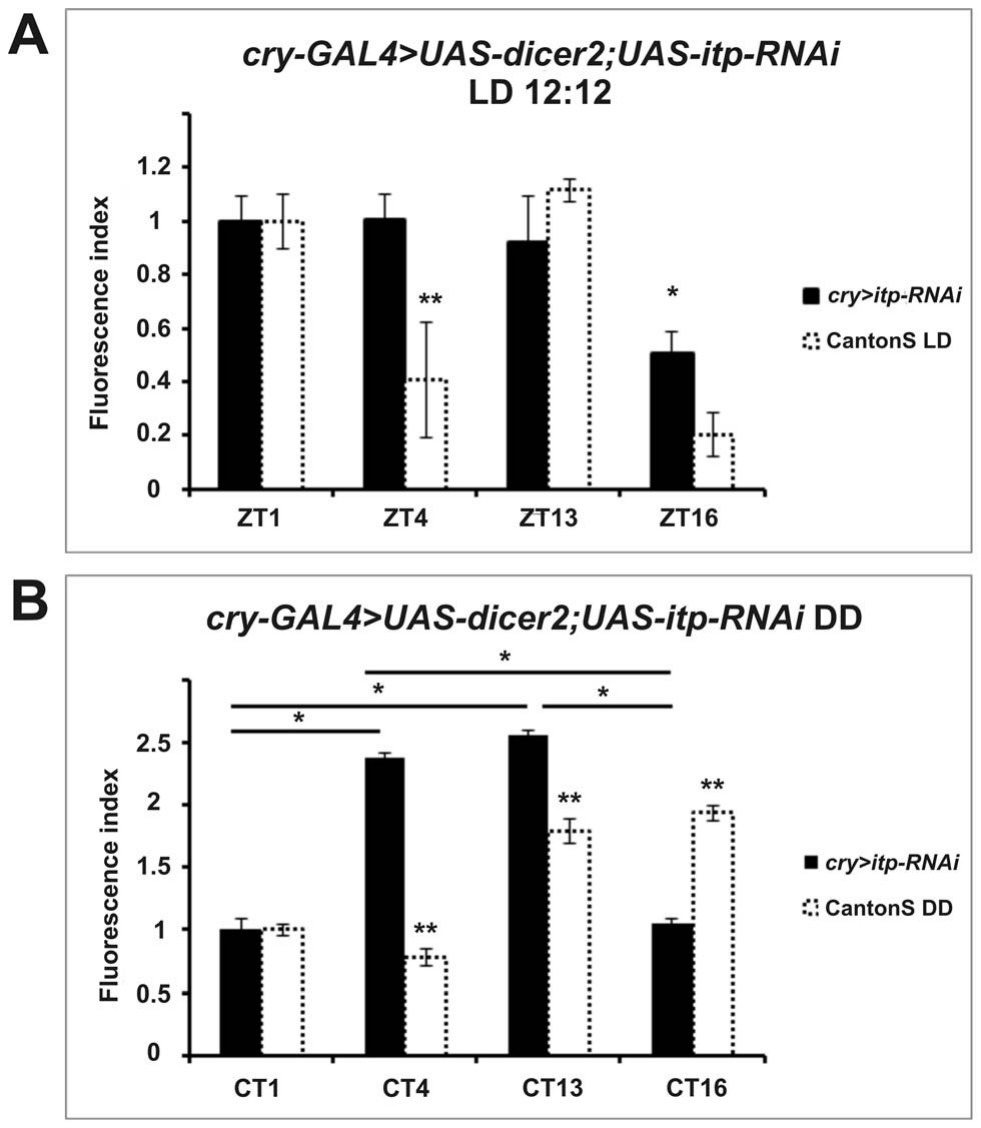
Pattern of ATPα immunoreactivity in flies with reduced ITP in CRY expressing cells under LD (A) and DD (B). The fluorescence index ± SE is shown as a function of time. (A) Under LD conditions the immunofluorescence index was high at ZT1, ZT4 and ZT13. It was lowered by 47.7% at ZT16. There were statistically significant differences between ZT16 and the other time points. (B) Under DD high immunosignal levels were observed at CT4 and CT13. There was a reduction of about 60% at the other time points There were statistically significant differences between CT1 and CT4, CT1 and CT13, CT16 and CT4, CT16 and CT13. Parametric ANOVA Tukey's test; p<0.05. The two stars symbols indicate statistically significant differences between the experimental strains and CantonS controls at different time points.

Finally, we downregulated ITP via RNAi in *cry* expressing cells (*cry-GAL4>UAS-dicer2, UAS-itp-RNAi*). Unlike controls, these flies failed to display ITP immunoreactivity in the lamina, which is derived from the ITP positive projections of the 5^th^ s-LN_v_ (Figure S2), and showed a dramatic impact on α-subunit immunoreactivity (Figures 8 and S3). Under LD, the immunosignal was constantly high except at ZT16 when its intensity dropped to approximately half that of other values (Figure 8A). Therefore under entrainment conditions it seems that ITP is required for decreasing α-subunit immunolabeling in the middle of the day (ZT4). In DD the rhythm was also unimodal but higher levels of immunofluorescence were seen at CT4 and CT13, which correspond to a shift of several hours compared to wild-type flies (peaks at CT13 and CT16, Figure 8B).

## Discussion

In all Diptera analysed thus far the first optic neuropil, the lamina, is a site of pronounced circadian plasticity where rhythmic changes in shape and size occur in interneurons and glia [38]. Morphological changes might derive, at least in part, from the modulation of the Na^+^/K^+^-ATPase, a major cellular pump that is able to import 2 K^+^ and export 3 Na^+^ for every ATP hydrolyzed. The sodium/potassium pump is under circadian regulation, as evident from the daily rhythms in immunodetection that have been described for the catalytic α subunit, ATPα, in neurons and glia in the optic lobe of *D. melanogaster* [21]. In this study we have confirmed that robust rhythmic changes of ATPα immunoreactivity occur in glial cells under LD and DD. Interestingly, circadian variation (low and high immunosignal during the subjective day and night, respectively) was further modulated by the light-dark cycle, resulting in a bimodal pattern with peak values at the times of light switch (ZT1 and ZT13, Figures 1 and 2). This correlates with the bimodal rhythms of locomotor activity and of size change of L1 and L2 monopolar cell axons in the lamina [15]. Furthermore, circadian regulation was abolished in *per^01^* [21] and *tim^01^* mutants, validating its rhythmic credentials (Figure 3A).

We then asked how light information is transmitted to the lamina glia, whether their own endogenous clock or rhythmic information from other clock cells is relevant for ATPα rhythmicity and finally which role, if any, is played by neuromodulatory peptides that are able to signal to the lamina, such as PDF and ITP.

The CRY protein is generally important for circadian photoreception as it is able to influence light-dependent locomotor activity phenotypes and to trigger downstream signaling after exposure to light [3], [39], [40]. There are two modes of action described thus far for light-activated CRY; one is seemingly cell autonomous and based upon its competence to promote the degradation of the key circadian protein TIM [39]; the other involves cellular cross-talk since CRY has been shown to increase neuronal firing after exposure to light [40]. Complete removal of CRY (*cry^01^* mutants) resulted in loss of LD modulation in the ATPαimmunoreactivity profile. However rhythmicity was retained, resulting in a pattern similar to that described for wild-type flies in DD (Figure 2B) also under light entrainment (Figure 4A). Reintroducing CRY *via* the UAS/GAL4 system rescued bimodality under LD (Figure 4C), further demonstrating that CRY is indeed involved in such regulation. To distinguish which function of CRY – cell-autonomous or cross-talk – is predominant in the lamina we used RNA interference to downregulate CRY expression in photoreceptors or glia, respectively (Figure S1). In both cases (Figure 5A, B) the bimodality of the immunosignal was retained. However, *repo-GAL4>UAS-cry-RNAi* flies showed a clear reduction in amplitude, suggesting that the cell-autonomous function of CRY is particularly important for the robustness of ATPα cycling in the lamina glia cells. We cannot, however, rule out the importance of CRY mediated cellular cross-talk as RNAi downregulated *cry* expression only by 36% (Figure S1).

We then considered whether intrinsic cellular rhythms or rhythmic input are important for ATPα immunoreactivity cycles in the lamina glia. As before we targeted photoreceptors and glia, this time overexpressing CYCΔ, a dominant negative form of CYC that is able to compromise the clock mechanism cell-autonomously [41] (Figure 6A, B). We were surprised to discover a milder phenotype (a reduction in the amplitude of the cycling of ATPα immunostaining) when interfering with the negative feedback directly in glia rather than in photoreceptors. The latter resulted in a profound alteration of the rhythmic profile such that a trough in immunofluorescence was detected at ZT4 only. While we cannot rule out that the negative feedback mechanism in photoreceptors might be more sensitive to the inhibitory action of CYCΔthan that in glia, nevertheless the profound effects we detected following manipulation of one of the main sources of rhythmic input to the lamina strongly suggests that signaling to reconcile rhythmic input with the endogenous feedback loop is a main constitutive element of the clock of glial cells. Moreover this result suggests that input from the retina photoreceptors may be responsible for the reduction of anti-ATPα immunoreactivity in the middle of the night. Our observations on the importance of the retina in regulating lamina rhythms add to previous results showing that photoreceptors modulate the rhythmic changes in axon size of L1 and L2 monopolar cells of the lamina [13], [14]. Here and in previous work we have also shown that glial cells contribute to maintaining lamina rhythms [42]. However, their effect seems weaker than that of photoreceptors and clock neurons, perhaps because of their lower clock gene expression [43].

The lamina expresses the PDF receptor [11] and is innervated by ITP-immunoreactive fibers originating from the 5^th^ s-LN_v_ [7] and therefore we examined whether these two neuropeptides are involved in the cycling of ATPα. In *Pdf^0^* flies, the pattern of ATPα immunoreactivity was the same in both LD and DD, showing one low-amplitude (but still significant) peak at ZT13 and CT13, respectively. We conclude that PDF is not required for ATPαcycling under DD (due to the peak at CT13) but intervenes to regulate the amplitude and the phase of the rhythm; in wild-type flies in DD, higher immunoreactivity was first detected at CT13 but persisted also at CT16, while it immediately dropped after CT13 in *Pdf^0^* mutants (Figures 2B and 7B). The bimodal pattern was re-established in LD after restoring PDF expression *via* the UAS/GAL4 system (Figure 7A, B, C). This suggests that PDF signaling is required for the peak of immunoreactivity at ZT1, which seemingly correlates with the time of higher PDF release [44]. Interestingly, expression of the pro-apoptotic gene *hid* in the PDF producing LN_v_s (*Pdf-GAL4*>*UAS-hid*), resulted in complete loss of rhythmic immunoreactivity in LD, and a more severe phenotype than that observed in *Pdf^0^* mutants. Our interpretation is that the LN_v_s probably influence the lamina *via* additional PDF-independent mechanisms.

We recently discovered ITP-immunoreactive processes in the distal lamina of *Drosophila*, which derive from the CRY-positive and PDF-negative 5^th^ s-LN_v_ [7]. Using a *cry-GAL4* driver we directed the expression of *UAS-itp-RNAi* (and of *UAS-dicer2* to help the RNAi mechanism) to reduce ITP levels in the lamina. We observed lack of ITP-immunofluorescence in the projections of the 5^th^ s-LN_v_ (Figure S2) and abnormal, albeit rhythmic, patterns of ATPα immunoreactivity in glia cells. Under DD (Figure 8B) the cycling of the immunofluorescence signal was advanced by several hours compared to wild-type flies. Under LD (Figure 8A), the immunofluorescence was always high except at ZT16, suggesting that appropriate ITP signaling is required for the reduction in anti-ATPα immunoreactivity observed in the middle of the day. Notably, a reduction in ITP expression via RNAi was reported to result in a longer period of locomotor activity [45]. This observation, in addition to ours, suggests a general role for ITP in circadian regulation. We have therefore unveiled a role for ITP in the nervous system; prior to this the only recognized role for ITP was that of an anti-diuretic factor in locust [46].

## Conclusions

We have described a complex regulation of the rhythm of abundance of ATPα measured as a cycle in the immunoreactivity of this protein in glial cells of the lamina. Since the catalytic subunit of the sodium pump is crucial for its activity, changes in the level of the α subunit likely reflect changes in the activity of the pump [47]. The cyclical activity of the sodium pump may then regulate the excitability of neurons in the brain either directly or indirectly via glial cells [48]. Furthermore, this rhythmicity may also constitute an energy saving mechanism that operates during sleep, which in flies occurs in the middle of the day and at night. In fact, the sodium pump is the primary energy consumer in the brain.

Some types of glial cells, including the epithelial glia of the lamina, express clock genes and function as circadian oscillators [49]. For instance, the lamina glia not only takes part in the metabolism of histamine, a neurotransmitter of the retina photoreceptors, but also regulates the rhythmic size change of the axons of the L1 and L2 monopolar cells [16]. Modulation of the Na^+^/K^+^-ATPase activity is a prominent factor of this regulation [50]. Indeed, we observed the lowest immunoreactivity, which we extrapolate as the lowest levels of activity of the Na^+^/K^+^-ATPase, at ZT4 and ZT16, which corresponds to the time when the L1 and L2 interneurons in the lamina are shrank [15] and when the level of the presynaptic protein BRUCHPILOT (BRP) is minimal in the retina photoreceptors [42]. A diurnal modulation of the activity of the Na^+^/K^+^-ATPase has also been found in the SCN of rat [51]. We suggest that the Na^+^/K^+^-ATPase is a universal key regulator of the clock-controlled plasticity of the brain.

## Supporting Information

Figure S1 **Reduction of** ***cry*** **mRNA in dissected retinas of** ***gmr-GAL4>UAS-cry-RNAi*** **flies.** The expression of *UAS-cry-RNAi* in photoreceptor cells using the *gmr-GAL4* driver resulted in a reduction of 36% in *cry* mRNA compared to the *gmr-GAL4* driver control (set to 1). Average normalized mRNA levels (± SE) for *cry* are shown. Quantification was carried out by reverse transcription real time PCR as described below. Thirty individuals were used from each of the following strains, *CantonS*, *gmr-GAL4*, *UAS*-*cry-RNAi* and *gmr-GAL4>UAS-cry-RNAi*. Retinas were cut off manually at ZT1 and total RNA was isolated using NucleoSpin RNA XS kit (Macherey-Nagel Germany) according to the manufacturer's protocol. 2 μg of total RNA was used for reverse transcription using a poly-T oligo and SuperScriptIII transcriptase (Invitrogen). The resulting cDNA was diluted 1∶8 and then used for quantitative PCR. TaqMan Gene Expression Assays labeled with 6′-FAM (Applied Biosystems) chemistry and 7500 Fast Real-Time PCR System (Applied Biosystems) were used to run reaction and analyse data. For *cry* gene mRNA assay and for *Ribosomal protein 32* (*rpl32*) as a reference gene, the TaqMan probes Dm02149911_m1 and Dm02151827_g1, respectively were used. Amplification reactions were performed in triplicate and repeated (biological replicates) at least 3 times. Data were collected as raw C_T_ values and analysed using the 2^−ΔΔCT^ method [52]. We observed similar levels of *cry* mRNA in *CantonS* (not shown), *UAS*-c*ry-RNAi* (not shown) and *gmr-GAL4* flies. In *gmr-GAL4>UAS-cry-RNAi* flies *cry* mRNA levels were reduced by 36%.
(TIF)

Figure S2 **ITP immunostaining in the lamina of CantonS (A) and** ***cry-GAL4>UAS-itp-RNAi*** **(B) flies.** **(**A**)** In CantonS flies processes from the 5^th^ s-LN_v_ that innervate the lamina (arrows) are labeled with anti-ITP serum (rabbit, 1∶1000; kindly donated by Dr. N. Audsley). (B) In *cry-GAL4>UAS-itp-RNAi* flies no ITP immunoreactivity was visible in the lamina. LA-lamina, ME-medulla, RE-retina.
(TIF)

Figure S3 **Wild type pattern of ATPα immunoreactivity in** ***UAS*** **-** ***itp-RNAi*** (**A) and** ***cry-GAL4*** **(B) control flies under LD.** The fluorescence index ± SE is shown as a function of time. (A) In UAS-*itp-RNAi* flies there were statistically significant differences between ZT1 and ZT4, ZT1 and ZT16, ZT13 and ZT4, ZT13 and ZT16. The fluorescence index peaked at ZT13 and was reduced by 60% at ZT4 and by 66% at ZT16. (B) A similar pattern was observed for *cry-GAL4* flies. The immunosignal was maximal at ZT13, and was reduced by 69.2% at ZT4 and by 63.8% at ZT16. Parametric ANOVA Tukey's test; p<0.05. We were unable to perform a rescue experiment of *itp* expression. However the pattern of anti-ATPα immunoreactivity in the lamina of *cry*-*GAL4* and *UAS*-*itp-RNAi* flies was the same as for CantonS (hatched lines).
(TIF)
