# Supplementary figures and images for: Circadian Regulation of the Na+/K+-Atpase Alpha Subunit in the Visual System Is Mediated by the Pacemaker and by Retina Photoreceptors in Drosophila Melanogaster
Source: PLoS One. 2013 Sep 10;8(9):e73690. doi: 10.1371/journal.pone.0073690 (PMC3769360; doi:10.1371/journal.pone.0073690)

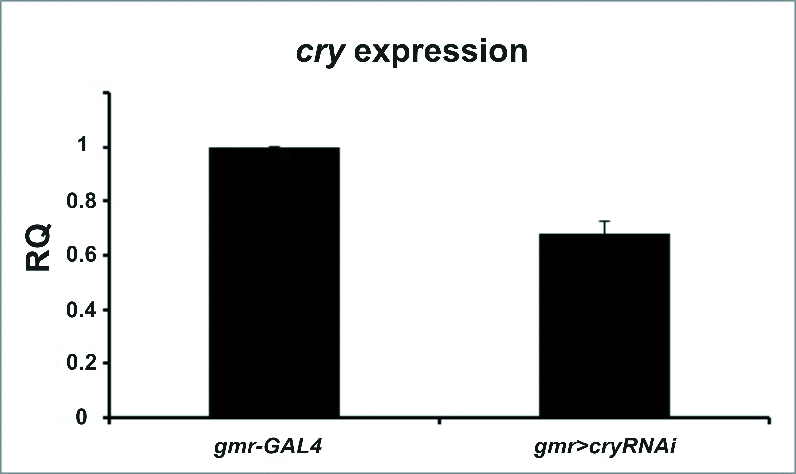

Supplement: Figure S1 — Reduction of cry mRNA in dissected retinas of gmr-GAL4>UAS-cry-RNAi flies. The expression of UAS-cry-RNAi in photoreceptor cells using the gmr-GAL4 driver resulted in a reduction of 36% in cry mRNA compared to the gmr-GAL4 driver control (set to 1). Average normalized mRNA levels (± SE) for cry are shown. Quantification was carried out by reverse transcription real time PCR as described below. Thirty individuals were used from each of the following strains, CantonS, gmr-GAL4, UAS-cry-RNAi and gmr-GAL4>UAS-cry-RNAi. Retinas were cut off manually at ZT1 and total RNA was isolated using NucleoSpin RNA XS kit (Macherey-Nagel Germany) according to the manufacturer's protocol. 2 μg of total RNA was used for reverse transcription using a poly-T oligo and SuperScriptIII transcriptase (Invitrogen). The resulting cDNA was diluted 1∶8 and then used for quantitative PCR. TaqMan Gene Expression Assays labeled with 6′-FAM (Applied Biosystems) chemistry and 7500 Fast Real-Time PCR System (Applied Biosystems) were used to run reaction and analyse data. For cry gene mRNA assay and for Ribosomal protein 32 (rpl32) as a reference gene, the TaqMan probes Dm02149911_m1 and Dm02151827_g1, respectively were used. Amplification reactions were performed in triplicate and repeated (biological replicates) at least 3 times. Data were collected as raw CT values and analysed using the 2−ΔΔCT method [52]. We observed similar levels of cry mRNA in CantonS (not shown), UAS-cry-RNAi (not shown) and gmr-GAL4 flies. In gmr-GAL4>UAS-cry-RNAi flies cry mRNA levels were reduced by 36%. (TIF) [file pone.0073690.s001.tif]

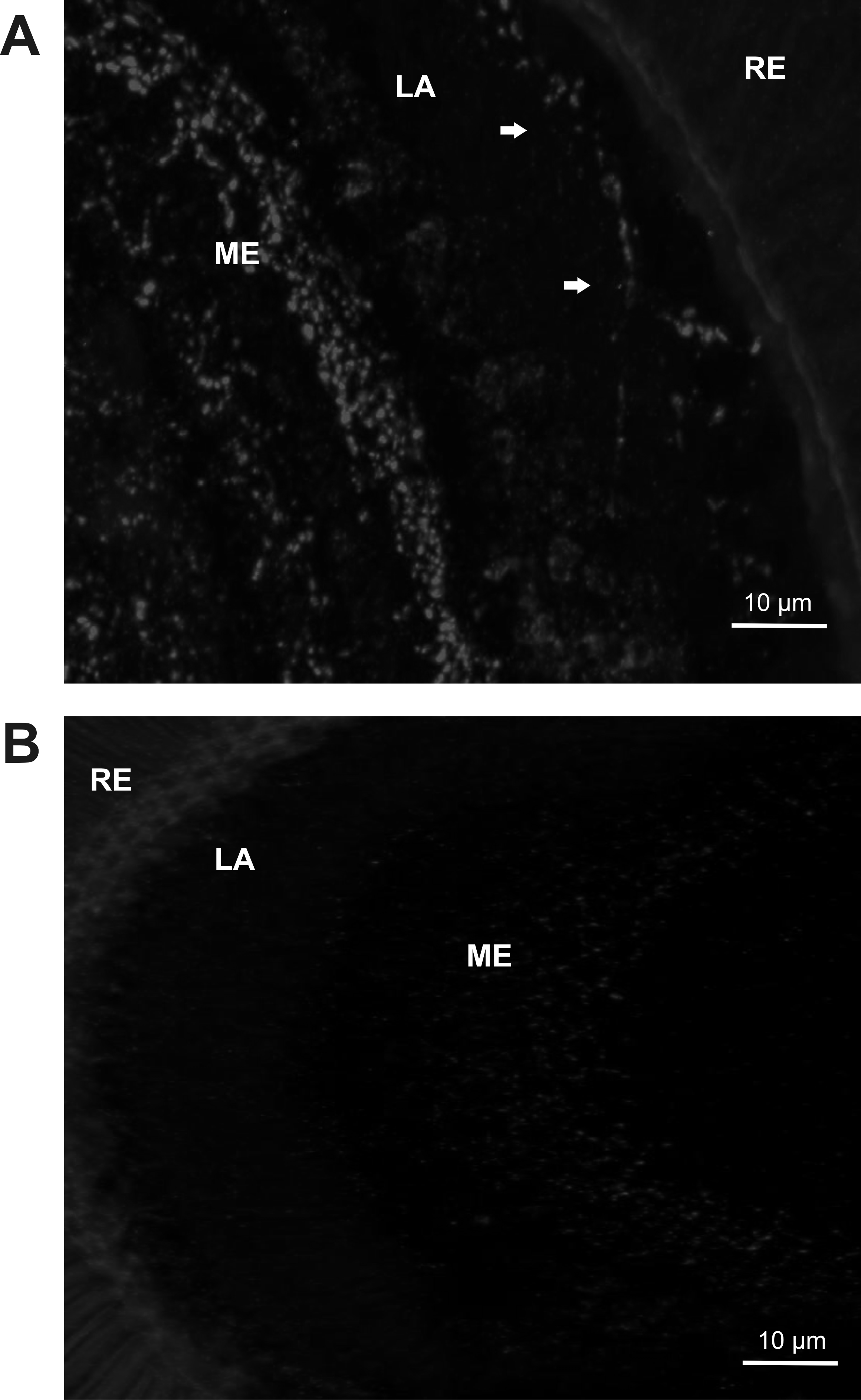

Supplement: Figure S2 — ITP immunostaining in the lamina of CantonS (A) and cry-GAL4>UAS-itp-RNAi (B) flies. (A) In CantonS flies processes from the 5th s-LNv that innervate the lamina (arrows) are labeled with anti-ITP serum (rabbit, 1∶1000; kindly donated by Dr. N. Audsley). (B) In cry-GAL4>UAS-itp-RNAi flies no ITP immunoreactivity was visible in the lamina. LA-lamina, ME-medulla, RE-retina. (TIF) [file pone.0073690.s002.tif]

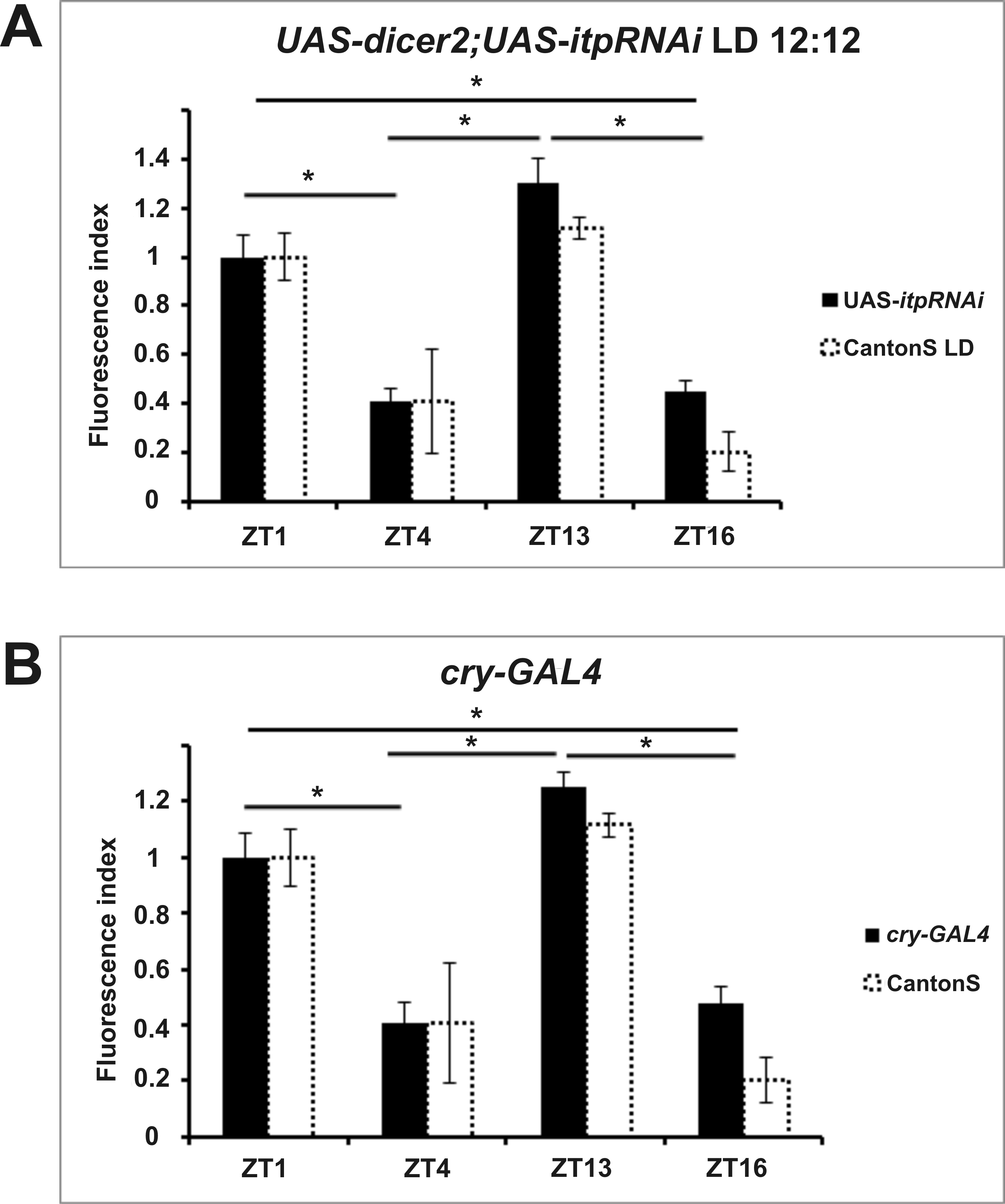

Supplement: Figure S3 — Wild type pattern of ATPα immunoreactivity in UAS - itp-RNAi (A) and cry-GAL4 (B) control flies under LD. The fluorescence index ± SE is shown as a function of time. (A) In UAS-itp-RNAi flies there were statistically significant differences between ZT1 and ZT4, ZT1 and ZT16, ZT13 and ZT4, ZT13 and ZT16. The fluorescence index peaked at ZT13 and was reduced by 60% at ZT4 and by 66% at ZT16. (B) A similar pattern was observed for cry-GAL4 flies. The immunosignal was maximal at ZT13, and was reduced by 69.2% at ZT4 and by 63.8% at ZT16. Parametric ANOVA Tukey's test; p<0.05. We were unable to perform a rescue experiment of itp expression. However the pattern of anti-ATPα immunoreactivity in the lamina of cry-GAL4 and UAS-itp-RNAi flies was the same as for CantonS (hatched lines). (TIF) [file pone.0073690.s003.tif]
